# Long-term survival and phenotypic expansion in siblings with generalized arterial calcification of infancy

**DOI:** 10.1210/jcemcr/luag091

**Published:** 2026-05-14

**Authors:** Ortal Resnick, Ambika Ashraf

**Affiliations:** Division of Pediatric Endocrinology and Diabetes, University of Alabama at Birmingham, Birmingham, AL 35233, USA; Division of Pediatric Endocrinology and Diabetes, University of Alabama at Birmingham, Birmingham, AL 35233, USA

**Keywords:** GACI, vascular calcification, hypophosphatemic rickets, bisphosphonate, fibroblast growth factor 23, pseudoxanthoma elasticum

## Abstract

Generalized arterial calcification of infancy (GACI) is a rare, autosomal recessive disorder caused by pathogenic variants in *ENPP1* or *ABCC6*. While typically fatal in infancy, survival into childhood is increasingly recognized. We report a family with 3 affected siblings homozygous for an *ENPP1* variant (c.*1367G*  *>*  *A*, p.*Arg456Gln*). The oldest died in infancy, the surviving 2 received early bisphosphonate therapy. Both survivors demonstrate persistent vascular calcifications, early-onset pseudoxanthoma elasticum (PXE)-like skin lesions, and chronic hypophosphatemia without radiographic rickets. Uniquely, this report contrasts the clinical course of a late preterm sibling against a sibling born extremely premature. Additional findings include auricular cartilage, renal and retinal calcifications, highlighting the systemic nature of ENPP1 deficiency. Our report expands the phenotypic spectrum of *ENPP1*-related GACI.

## Introduction

Generalized arterial calcification of infancy (GACI) provides a rare model of dysregulated pyrophosphate and phosphate metabolism, with profound implications for vascular mineralization, fibroblast growth factor 23 (FGF23) biology, and long-term skeletal outcomes.

GACI is an extremely rare autosomal recessive disorder characterized by diffuse calcification and stenosis of large and medium-sized arteries, typically presenting in the prenatal or early neonatal period. The estimated incidence is 1 in 200 000 pregnancies, with 1 in 1 000 000 live births [1, 2]. GACI is caused by biallelic *ENPP1* or *ABCC6* variants, leading to a deficiency of inorganic pyrophosphate (PPi), a potent inhibitor of pathological tissue mineralization. The resultant vascular calcifications frequently lead to severe cardiovascular compromise, including systemic hypertension, respiratory distress, cardiomyopathy, and high neonatal mortality. While historical mortality rates range from 55% to 85% in the first 6 months [1, 3], recent cohorts suggest survival is improving, necessitating a better understanding of the long-term phenotype. Among these survivors, mortality has been reported at approximately 7% primarily due to cardiac or neurological complications [3]. In survivors, several reports describe vascular calcifications that have regressed or even resolved entirely [4-7]. In addition to vascular complications, GACI survivors may develop late-onset manifestations such as pseudoxanthoma elasticum (PXE)-like skin changes, nephrocalcinosis, and autosomal recessive hypophosphatemic rickets type 2 (ARHR2).

PXE-like cutaneous findings typically manifest during childhood, with onset between ages 2 and 43 years [2]. The classic form of PXE is caused by mutations in the *ABCC6* gene; however, it has also been described with mutations in the *ENPP1* gene [8-11], ie, PXE-like. It has been suggested that GACI and PXE represent a spectrum of ectopic mineralization disorders rather than entirely distinct entities, with both conditions linked to reduced PPi. Recent large-scale updates on *ENPP1* mutations have expanded the clinical spectrum to include cutaneous hypopigmentation and punctate keratoderma, yet data on long-term progression remain vital [12].

Nephrocalcinosis most commonly appears during infancy. Medullary nephrocalcinosis is typically observed in patients treated for hypophosphatemia, whereas cortical nephrocalcinosis can occur in untreated individuals and is thought to result from renal ischemia secondary to vascular compromise [1]. Hypertension reported prevalence varies across studies, ranging from 12% to 33% to as high as 60% [1, 2]. Hypertension likely reflects arterial stenosis and reduced renal perfusion, and may persist despite regression of vascular calcifications [7].

Skeletal and biochemical phenotypes evolve with age. Infants typically display normal phosphate and normal-to-low FGF23 during the calcification phase; survivors often transition to an ARHR2 phenotype. This is characterized by elevated FGF23, renal phosphate wasting, hypophosphatemia (average onset 1.6 years), and rickets by age 14, and elevated serum alkaline phosphatase (ALK-P) [2]. Parathyroid hormone (PTH) is normal or slightly elevated while serum calcium and 1,25-OH-D3 levels are typically within the normal range [13]. Management guidelines remain controversial, especially regarding the duration and safety of bisphosphonate therapy [14].

Here, we describe siblings carrying a rare *ENPP1* variant associated with GACI.

## Case presentation

The parents are healthy and non-consanguineous. The family history includes one miscarriage, a sister who died at 4 months of age from GACI, and 2 surviving siblings affected by the condition. No parental medical conditions associated with connective tissue, cardiovascular, or metabolic disorders have been reported.

Sibling 1, a boy, was born late preterm with respiratory distress syndrome. Extensive vascular calcifications were noted on evaluation at 2 days of age, prompting clinical concern for GACI. Sibling 2, a girl, was born extremely premature after a pregnancy complicated by fetal hydrops and pericardial effusion. Given the family history, she was clinically presumed to have GACI shortly after birth.

A comparative overview of their clinical features is summarized in Table 1. Neither sibling reported chronic pain or muscle weakness; renal function and neurodevelopment remained age-appropriate. Although ophthalmologic evaluation identified retinal vascular involvement in both siblings, no significant functional visual impairment related to vascular calcification was reported. For sibling 1, aside from a single traumatic fracture, there was no history of recurrent fractures or skeletal deformities, and radiographs showed no evidence of rickets. For sibling 2, she has orthopedic limitations related to hip dysplasia and prior surgical interventions and had no history of fractures.

**Table 1:** Clinical, biochemical, and imaging features of 2 siblings with ENPP1-related GACI

Feature	Sibling 1 (Male, 12y)	Sibling 2 (Female, 8y)
*ENPP1* variant	Homozygous c.*1367G* *>* *A* (p.*Arg456Gln*)	Homozygous c.*1367G* *>* *A* (p.*Arg456Gln*)
GA, BW	GA 33 weeks; BW 2.098 kg	GA 25 weeks; BW 0.91 kg
Age at diagnosis	2 days of life	1 day of life
Initial clinical presentation	Pulmonary hypertension; RDS	Fetal hydrops; Pericardial effusion; Pulmonary hypertension; Hyperbilirubinemia; RDS
Bisphosphonate therapy	Pamidronate started in first week of life (0.1 mg/kg/week) until 6 months; transitioned to weekly risedronate 1 mg/kg/week from 6 months to 2 years; risedronate 0.5 mg/kg/week from 2 to 7 years	Pamidronate started in first week of life (0.1 mg/kg/week) for 3 months; transitioned to weekly risedronate 1 mg/kg/week from 3 months to 3 years 7 months
Initial vascular calcifications	Aorta, pulmonary arteries, coronary arteries, SMA, celiac, splenic, renal, iliac arteries	Echobright great vessels on admission echocardiogram, including main pulmonary artery and aortic arch
CT*^a^* calcifications	Left circumflex coronary artery, proximal descending aorta, right renal artery origin, bilateral common iliac arteries,	Aortic root, ascending and thoracic aorta, left circumflex coronary artery, SMA origin and branches, left common iliac artery,
Renal findings	US—calcifications predominately in the renal medullary pyramids CT—hyperdensities at the corticomedullary junctions	US—Diffuse cortical and medullary calcifications CT—Cortical calcifications
Other CT*^a^* findings	Auricular cartilage, pancreatic head	Auricular cartilage
PXE-like skin changes	Yes, onset 7 years; progressive	Yes, onset 2 years; progressive
Hypophosphatemia	Present from 6 months	Present from birth
Intact FGF23 levels (<52 ng/L)	Elevated (75-81 ng/L)	Elevated or high-normal (43-69 ng/L)
TRP (80-95%)	Improved from 16.5% to 96.9%	Normal 93.6-96.1%
Bone density*^a^*	Total *Z*-scores −0.7 (age 12)	Total *Z*-scores −0.9 (age 8)
Radiographs*^a^*	No evidence of rickets	No evidence of rickets
Orthopedic complications	None	Right hip dysplasia; surgery
Hearing	Normal	Mild low-frequency conductive hearing loss in left ear
Ophthalmologic findings	Partial retinal artery occlusion, vascular calcifications	Partial retinal artery occlusion, vascular calcifications

Reference ranges: intact FGF23 < 52 ng/L.

Abbreviations: ALK-P, alkaline phosphatase; BW, birth weight; CT, computed tomography; FGF23, fibroblast growth factor 23; GA, gestational age; PXE, pseudoxanthoma elasticum; RDS, respiratory distress syndrome; SMA, superior mesenteric artery; TRP, tubular reabsorption of phosphate; US, ultrasound.

^*a*^CT imaging, bone density assessment, and radiographs were obtained at age 12 years for sibling 1 and at age 8 years for sibling 2.

## Diagnostic assessment

Genetic testing confirmed that all 3 affected siblings were homozygous for the *ENPP1* c.1367G > A (p.Arg456Gln) missense variant. Genetic testing in the sibling who died in infancy was performed at an outside institution prior to the birth of sibling 1, however, details regarding the timing of testing and the specific source of DNA were not available. The parents were not genetically tested, but are obligate carriers based on having 3 affected children with an autosomal recessive condition. Several additional heterozygous variants of uncertain significance were identified in *LTBP3*, *SMAD4, NOTCH2*, and *SETBP1* but were considered noncontributory given the inheritance patterns and absence of any related parental phenotype.

**Figure 1:**
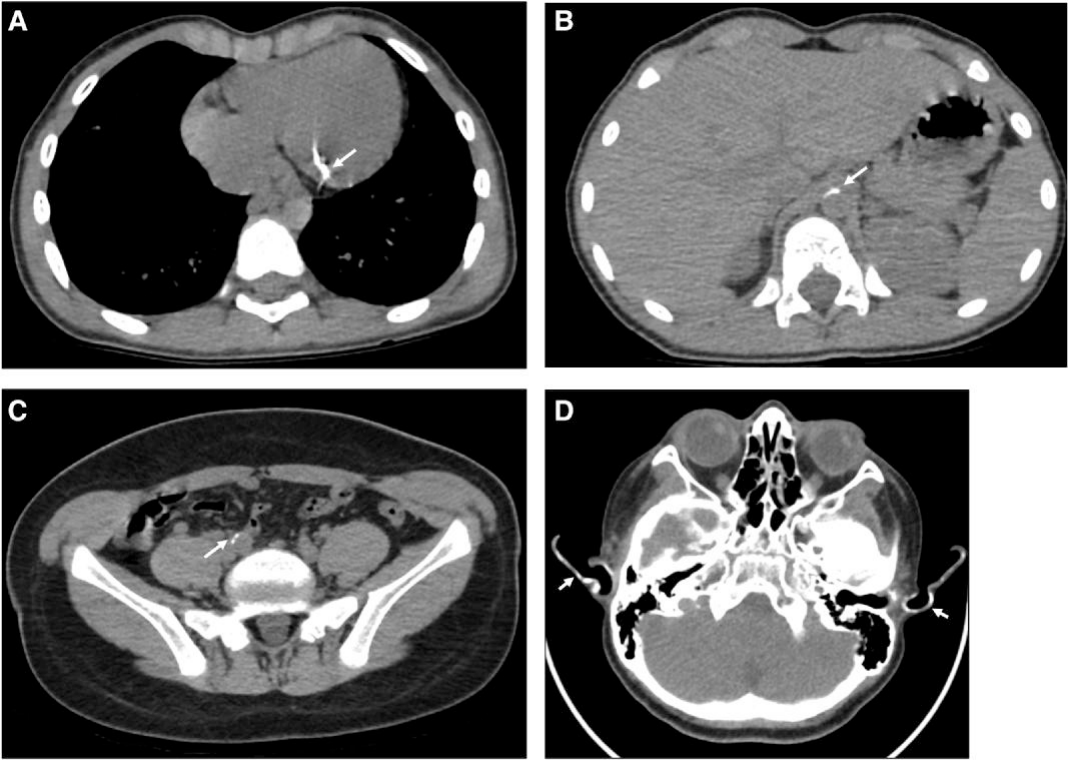
CT imaging in siblings with ENPP1-related generalized arterial calcification of infancy (GACI). Panel (A) demonstrates calcification of the left circumflex coronary artery in sibling 2. Panel (B) shows calcification at the origins of the superior mesenteric and celiac arteries in sibling 2. Panel (C) shows calcification at the origin of the right renal artery in sibling 1. Panel (D) demonstrates calcification of the auricular cartilage in sibling 1.

**Figure 2:**
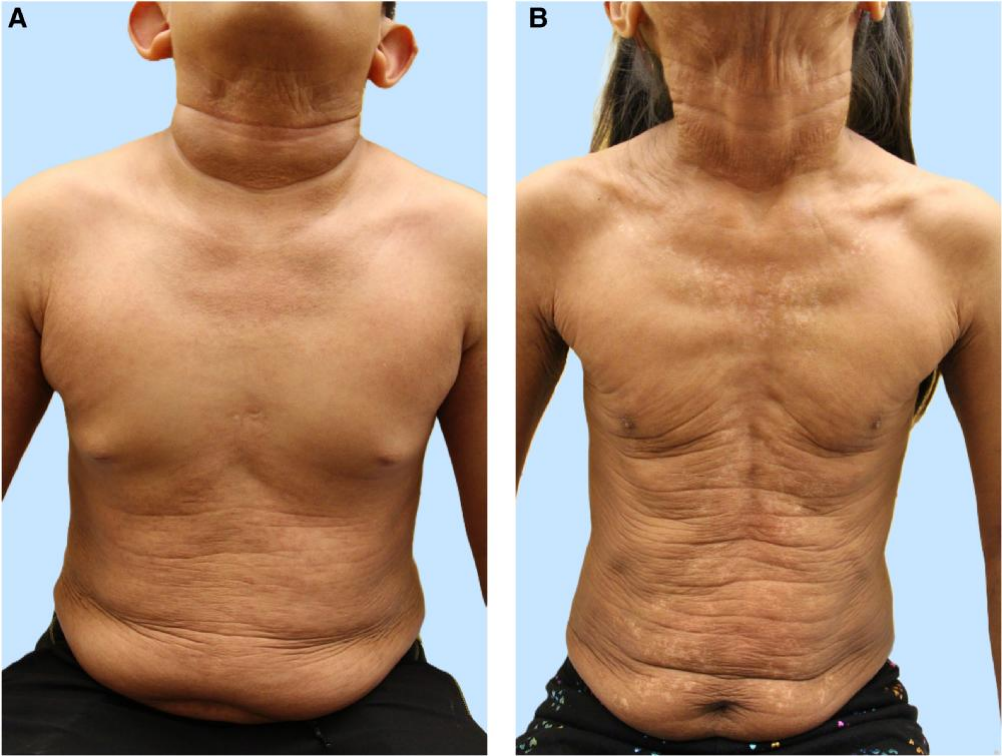
Pseudoxanthoma elasticum-like skin changes in siblings with ENPP1-related GACI. Panel (A) demonstrates redundant skin folds on the neck, abdomen, and axillae, with earlobe involvement due to auricular cartilage calcification, resulting in outwardly projected earlobes in sibling 1 at age 12 years. Panel (B) shows diffuse skin laxity and redundant folds over the neck, abdomen, and axillae in sibling 2 at age 8 years.

For sibling 1, diagnostic evaluation in infancy included head ultrasounds without evidence of intracranial calcification. Cardiac assessment between 4 and 6 years revealed thickened, echo-bright mitral and aortic valve leaflets with progression to moderate mitral regurgitation by 6.5 years of age. A nongated chest computed tomography (CT) angiogram at 12 years showed persistent vascular calcifications (Fig. 1C-1D). Abdominal ultrasound at 19 months demonstrated early nephrocalcinosis with diffuse arterial wall calcification. Over time, the appearance evolved from cortical and corticomedullary calcifications to predominantly medullary pyramidal echogenic foci by 7 years of age, consistent with findings on CT at 12 years. Renal Doppler studies at 9 years were normal. Laboratory evaluation showed persistently low serum phosphate 3.2-3.8 mg/dL (1.03-1.23 mmol/L; reference range 4.1-5.9 mg/dL [1.32-1.90 mmol/L]) with elevated intact FGF23, although tubular reabsorption of phosphate (TRP) improved markedly from 16.5% at age 7 to 96.9% (reference range 85-95%) at age 12. ALK-P gradually increased over childhood and reached 532 U/L at 12 years of age (reference range 141-460 U/L). Dermatologic examination revealed PXE-like cobblestone-textured skin lesions over the trunk and neck at age 7 (Fig. 2A).

For sibling 2, a head ultrasound at 1 month of age showed normal brain echotexture. Early echocardiography revealed echobright great vessel walls, moderate pericardial effusion, and moderate postductal aortic narrowing with diffuse abdominal aortic hypoplasia. A moderate patent ductus arteriosus was closed at 4 months, and infantile left ventricular hypertrophy and dilation resolved by 6 years of age. At 8 years, a nongated chest CT identified multiple vascular calcifications (Fig. 1A-1B). Renal ultrasounds were initially normal but progressed by age 3 to show increased echogenicity and linear calcifications, evolving into diffuse cortical and medullary nephrocalcinosis by age 8, consistent with cortical calcifications seen on CT. Laboratory evaluation revealed persistent hypophosphatemia (3.5-3.8 mg/dL [1.13-1.23 mmol/L]) with mildly elevated or high-normal intact FGF23 levels, while TRP remained preserved and ALK-P remained within the normal range. She developed PXE-like lesions at 2 years of age involving the abdomen, posterior neck, and lower back (Fig. 2B). Labial adhesions were documented beginning at age 3.

## Treatment

Both siblings received bisphosphonate therapy in infancy and early childhood (Table 1). Hypertension in both is managed with amlodipine. Orthopedic management for sibling 2 included treatment of right hip dysplasia, diagnosed at 11 months, with right hip internal fixation at age 7, followed by surgical adductor release for an adduction contracture at age 8. No phosphate supplementation was initiated because TRP was preserved in sibling 2, and increased gradually in sibling 1.

## Outcome and follow-up

At the last follow-up, sibling 1 (age 12) and sibling 2 (age 8) demonstrated stable hemodynamic status with preserved ventricular function. PXE-like skin findings continued to evolve, and both remain normotensive on amlodipine.

Sibling 2 has residual orthopedic limitations following hip surgeries but remains ambulatory.

## Discussion

This report describes the long-term clinical course of 2 siblings with ENPP1 deficiency who survived beyond infancy following early bisphosphonate therapy. To the best of our knowledge, only a limited reports mention *ENPP1* c.1367G > A variant (p.Arg456Gln), and none have provided detailed clinical descriptions [15, 16]. The p.Arg456Gln substitution affects a conserved residue within the catalytic phosphodiesterase domain and has been reported in a limited number of patients with pathogenic ENPP1-related disease [15].

Our report underscores the unusually early development of PXE-like skin changes, persistent vascular calcifications into late childhood, early childhood retinal artery involvement, and auricular cartilage calcification. Unlike prior reports describing regression of calcifications by age 5, our patients demonstrate persistence at ages 12 and 8 years [4-6].

Ectopic calcification in GACI results from reduced extracellular PPi. Unlike prior reports describing partial or complete regression of calcifications by age 3 to 5 years in survivors [4-6], our patients demonstrate persistent vascular calcifications at ages 12 and 8 years, respectively. Both siblings demonstrated persistent renal calcifications on imaging. The natural history of nephrocalcinosis in GACI is not well characterized, and there is no clear evidence that it resolves over time, even if vascular calcifications regress [1]. In sibling 2, the renal findings likely represent a “double hit” phenomenon: the genetic loss of PPi combined with the nephrotoxic events inherent to extreme preterm care.

Both siblings presented with hypophosphatemia much earlier than average (at birth and 6 months) [2]. Despite chronic hypophosphatemia, neither sibling developed radiographic evidence of rickets. Interestingly, the older sibling showed a unique improvement in TRP from 16.5% to 96.9% over 5 years, despite persistently elevated intact FGF23 (paradoxical renal FGF23 resistance). This unusual pattern suggests possible compensatory renal adaptation or post-translational FGF23 regulation, underscoring the need for continued monitoring for bone disease as these patients age.

Our patients also exhibited unusually early dermatologic and ophthalmologic manifestations. PXE-like lesions, classically associated with *ABCC6* mutations in the second decade of life, are increasingly linked to *ENPP1* deficiency [2, 9, 17]. Our patients presented with skin changes unusually early, at ages 2 and 7 years. Similarly, ocular involvement, specifically retinal artery occlusions and calcifications, was detected far earlier than the typical adult presentation [1]. While prematurity is a significant confounder for retinopathy in sibling 2, the presence of similar findings in the older sibling suggests that early ocular involvement may be an underrecognized feature of ENPP1 deficiency. Additionally, both siblings demonstrated auricular cartilage calcifications, further expanding the recognized clinical spectrum of chronic, multi-organ calcification in this condition [18].

Significant intrafamilial phenotypic variability is a hallmark of this report. Despite sharing the same genotype, the outcomes ranged from infantile death in the untreated oldest sister, to a relatively stable course in sibling 1, and significant orthopedic and dermatologic complications in sibling 2. One pregnancy ended in miscarriage; the genetic status of that fetus is unknown. This variability highlights the influence of modifier genes, environmental factors, and treatment timing. In this family, the extreme prematurity of sibling 2 can be a dominant modifier driving a more complex clinical course.

Management of GACI remains challenging due to a lack of consensus on therapy duration and definitive evidence of efficacy [1, 4, 19]. Bisphosphonates are used due to their structural similarity to PPi, theoretically inhibiting calcium phosphate crystal deposition. While animal studies show preventative effects, human studies regarding the reversal of established calcifications are mixed. However, a retrospective study suggested a survival benefit when therapy is initiated within the first 7 days of life [3]. In this family, the long-term survival of the 2 siblings treated within the first week of life, compared with their untreated sister who died at 4 months, supports the potential survival benefit of early bisphosphonate intervention. We discontinued therapy at ages 7 and 3.5 years, respectively, after calcification stabilization. We recognize the limitation of our small sample size (*n* = 2) and the well-described variability in clinical expression of ENPP1 deficiency, even among individuals carrying identical variants [15].

Looking forward, emerging therapies aimed at the underlying pathophysiology may facilitate survival and suppress systemic disease progression. Enzyme replacement therapy with recombinant ENPP1 (currently under investigation)holds promise but remain experimental.

## Learning points

- ENPP1 deficiency presents as a multi-organ disorder, including vascular calcifications, PXE-like skin changes, and hypophosphatemia.
- Survival does not equate resolution; vascular calcifications can persist long-term, underscoring the need for lifelong monitoring.
- Clinical presentation can vary dramatically within families, highlighting the importance of environmental modifiers.

## Contributors

All authors made individual contributions to the authorship. OR, AA: were involved in the diagnosis and management of the patients, literature review, and manuscript preparation. OR: primary manuscript drafting, table and figure preparation. AA: critical revision of the manuscript. All authors reviewed and approved the final draft.

## Data Availability

Original data generated and analyzed for this case report are included in this published article.

## Abbreviations

ALK-P: alkaline phosphatase
ARHR2: autosomal recessive hypophosphatemic rickets type 2
CT: computed tomography
FGF23: fibroblast growth factor 23
GACI: generalized arterial calcification of infancy
PPi: inorganic pyrophosphate
PTH: Parathyroid hormone
PXE: pseudoxanthoma elasticum
TRP: tubular reabsorption of phosphate
